# Hygroscopic effect of high clay-content shale under temperature and humidity conditions and its impact on mechanical properties

**DOI:** 10.1371/journal.pone.0319672

**Published:** 2025-03-07

**Authors:** Weisheng Zhao, Wei He, Lichao Hu, Zhaoan Wang

**Affiliations:** 1 School of Architectural Engineering, Neijiang Normal University, Neijiang, Sichuan, China; 2 State Key Laboratory of Intelligent Construction and Healthy Operation and Maintenance of Deep Underground Engineering, China University of Mining and Technology, Xuzhou, Jiangsu, China; Sichuan University of Science and Engineering, CHINA

## Abstract

High clay-content shale, containing hydrophilic clay minerals, is highly sensitive to environmental temperature and humidity. It readily absorbs moisture from the air, leading to increased water content and reduced mechanical strength, which poses challenges for underground structures, such as mining roadways, tunnels, and storage chambers. This study investigates the influence of temperature and humidity on the water content of high clay-content shale during its hygroscopic process and examines the evolution of its mechanical properties under variations in water content, aiming to reveal the effects of environmental temperature and humidity on the mechanical behavior of high clay-content shale. Hygroscopic experiments were conducted using a temperature and humidity chamber, with quartz sand as non-clay mineral control groups, and strength experiments were performed on reconstituted shale samples with varying water content. Results from the hygroscopic experiments showed that the equilibrium water content (EWC) of high clay-content shale decreases with lower humidity and higher temperature. When the humidity decreased from 100% RH to 80% RH, the average EWC dropped from 15.88% to 7.53%. Under high-humidity conditions (100% RH), the EWC decreased to 11.92% only after the temperature increased to 30°C. Within the experimental conditions, reducing humidity was found to be more effective than increasing temperature in reducing EWC. Based on the mechanical test results, reducing humidity can decrease the loss of uniaxial compressive strength (UCS) caused by moisture absorption from approximately 50% to 15.48%. The results indicate that humidity is the primary factor influencing the EWC and mechanical properties of high clay-content shale. Reducing humidity can significantly mitigate strength loss caused by moisture absorption, while increasing temperature plays a supplementary role. These findings provide a scientific basis for controlling temperature and humidity in underground engineering to enhance structural stability.

## 1. Introduction

High clay-content shale, due to its rich content of clay minerals and complex microstructure, is highly sensitive to environmental temperature and humidity, exhibiting significant hygroscopic properties [1–3]. During the hygroscopic process, the increase in moisture content significantly weakens its mechanical properties, often leading to instability in the surrounding rock of underground engineering projects, such as mine tunnels, subways, and energy storage chambers [4–9]. This instability can result in strength degradation, increased deformation, and even engineering failure, posing great challenges to construction and long-term maintenance. Although previous studies have focused on the effects of humidity on rock properties, the synergistic effects of temperature (especially in the normal temperature range) and humidity on the hygroscopic characteristics and mechanical properties of high clay-content shale have not been systematically revealed. Based on experimental methods, this study, for the first time, explores the coupled effects of temperature and humidity on the hygroscopic properties of high clay-content shale, examining the dynamic characteristics of moisture content changes over time and under varying temperature and humidity conditions, as well as the specific effects of moisture content changes on uniaxial compressive strength and elastic modulus. The findings not only enrich the theoretical understanding of the hygroscopic properties of high clay-content shale but also provide scientific evidence for optimizing environmental conditions in underground engineering (such as humidity control and temperature regulation).

Clay minerals, such as montmorillonite and illite, are highly sensitive to moisture and can rapidly absorb water from sources like groundwater, construction-induced infiltration, or atmospheric water vapor [10–13]. Among these sources, atmospheric water vapor is a primary contributor to moisture absorption by clay minerals, especially in high-humidity environments, where it can rapidly increase the rock’s water content [14,15]. This hygroscopic behavior leads to volume expansion of the rock and significantly weakens its mechanical properties [16,17].

The water content has a pronounced effect on the mechanical properties of clay-rich rocks. Research by, Hawkins & Mcconnell [18], Vasarhelyi [19], and Deng et al. [20], indicates that increased water content significantly reduces the compressive strength, shear strength, and elastic modulus of rocks. This degradation is attributed to the swelling effect of clay minerals when they absorb water. Expansive clays like montmorillonite and illite swell upon water absorption, destroying the microstructure of the rock and leading to a decline in mechanical properties. Chen et al. [21] used a universal testing machine and scanning electron microscopy to study the mechanical properties and microstructure of mudstone under different water contents, establishing a mechanical model for porous media. Cherblanc et al. [22] explored the mechanical weakening of limestone under varying water content, highlighting the number of montmorillonite layers as a controlling factor in water absorption capacity, which showed a linear relationship with strength loss. Wang et al. [23], through compression, Brazilian, and shear tests, investigated the mechanical characteristics of loess under different moisture levels, finding a clear linear relationship between mechanical properties and increased water content.

Environmental conditions such as temperature and humidity play a significant role in the evolution of water content and the mechanical properties of clay rocks. Regarding temperature, previous studies have focused on changes in rock mechanics under extreme temperature conditions. For instance, Bani et al. [24] examined the impact of freeze-thaw cycles on natural bentonite-modified asphalt concrete mixtures, while Bozbey et al. [25] investigated changes in the elastic modulus and frost resistance of lime-stabilized soil. Gbewade et al. [26] studied the mechanical properties of clay rocks under different temperatures (20-150°C), analyzing the variation in peak strength and the propagation of microcracks with temperature changes. Zhang et al. [27] described several physicochemical reactions in clay rocks under high temperatures (300-600°C) and their impact on production, analyzing the changes in physical and mechanical parameters of clay rocks under varying temperatures and providing the critical threshold range for thermal damage in rocks. Tian et al. [28] and Skrzypkowsk et al. [29] studied the physical and mechanical properties of clay rocks at 1000°C and 1200°C, respectively. However, relatively few studies have investigated the effects of temperature on rock water content within the moderate temperature range suitable for construction (e.g., 5-30°C).

Humidity, in conjunction with water content, is frequently discussed in geotechnical engineering. In many studies, “humidity” refers to the water content of geotechnical materials and describes its impact on mechanical properties, crack development, and durability [30–34]. Humidity refers to the mass of water vapor in the air, while relative humidity is the ratio of the actual water vapor content in the air to the maximum water vapor capacity at that temperature. Clay minerals (e.g., montmorillonite, illite, and kaolinite) can increase their water content by absorbing moisture from the air, leading to a decline in rock mechanical performance. For example, Nara et al. [35] investigated the fracture toughness of clay-bearing sandstone under different relative humidity conditions, finding that montmorillonite absorbs moisture under high humidity conditions, causing the rock to expand, lose strength, and experience crack propagation. Erguler et al. [14] monitored the water content variations in rocks with different clay contents under relative humidity levels of 90-100%. Wang et al. [36] examined the creep behavior of rock salt under 33%, 55%, and 77% relative humidity conditions, proposing a humidity-dependent creep law. Garcia-Fernandez et al. [15] observed that increased relative humidity caused a threefold decrease in the tensile strength of slate. Champiré et al. [37] noted that relative humidity affects the mechanical properties of compacted soil, leading to changes in the soil’s water content. However, studies on the temporal evolution of water content in geotechnical materials under varying relative humidity remain relatively scarce.

Therefore, this study focuses on high clay-content shale, taking into account the actual construction and operational environment of underground engineering projects, such as mine roadways and tunnels. It selects a temperature range (5°C-30°C) and relative humidity (40%RH-100%RH) as the research variables, with four levels set for each. To more accurately reflect the hygroscopic properties of the clay minerals in high clay-content shale, quartz sand samples (without clay minerals) are used as the control groups. Additionally, in the hygroscopic experiments, temperature and humidity conditions are kept constant, and all samples (including clay mineral samples and quartz samples) undergo three sets of parallel experiments to ensure the reliability of the experimental data. By studying the effect of temperature and humidity on moisture content changes during the hygroscopic process of high clay-content shale, and combining uniaxial compressive strength tests of reconstituted shale samples under different moisture content conditions, this study explores the changes in the mechanical properties of high clay-content shale after moisture absorption. The results not only provide experimental evidence for further revealing the impact mechanism of temperature and humidity variations on the structural stability of high clay-content shale in underground engineering, but also offer practical theoretical guidance for optimizing underground engineering environments, with significant practical value.

## 2. Materials and methods

### 2.1. Materials and experimental samples

The high clay-content shale used in this study was collected from the Cretaceous strata in Xilin Gol League, Inner Mongolia, with an average burial depth of approximately 300 meters. The shale is dark brown and rich in organic matter. XRD diffraction analysis of the shale (S1) revealed that clay minerals account for 51% of the total mineral composition, while non-clay minerals constitute 49%, with quartz comprising 41% of the total (Table 1). Due to its high clay mineral content, the rock sample exhibits significant susceptibility to weathering (S2). Upon exposure to water, the sample softens, disintegrates, and turns into mud rapidly (S3). Furthermore, it readily absorbs atmospheric moisture when exposed to air, leading to a significant reduction in its mechanical properties. This characteristic may negatively impact structural stability in underground engineering applications.

**Table 1:** Qualitative and quantitative analysis results of whole-rock minerals.

Mineral	Non-clay minerals			Clay minerals		
	Quartz	Albite	Microcline	Kaolinite	Illite	Illite-Smectite
**Content (%)**	41%	3%	5%	18%	2%	31%

**Fig 1:**
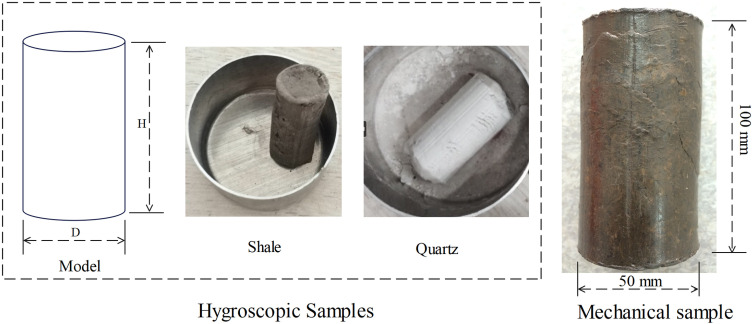
Representative experimental samples.

To investigate the effects of humidity and temperature on the hygroscopic behavior of clay minerals and its impact on the rock’s water content, we ground the original rock into a 200-mesh powder, added water, and mixed it to a plastic state to create hygroscopic samples (S4 and Fig 1). Simultaneously, quartz sand (SiO₂) was used as a representative of non-clay minerals, and similar hygroscopic samples were prepared following the same method to serve as control groups, thereby reflecting the hygroscopic characteristics of clay minerals within the rock.

In addition, to analyze the weakening of mechanical strength in rock samples due to changes in water content after hygroscopic absorption, we attempted to conduct mechanical experiments using standard-sized shale samples. However, due to the high clay mineral content and sensitivity to water, the rock exhibited low mechanical strength, and efforts to obtain φ50mm × 100mm standard cores through field drilling or laboratory preparation were unsuccessful, resulting in an RQD of 0. To address this, we employed a custom-designed reconstruction platform (S5) to simulate the diagenetic process of the rock at a burial depth of 300 meters using a four-stage graded loading approach. This approach successfully produced standard-sized experimental specimens (Fig 1), which were then utilized in experiments to evaluate mechanical performance. The reconstitution process of high clay-content shale is detailed in S5.

### 2.2. Hygroscopicity experiments

#### 2.2.1. Experimental scheme.

**Fig 2:**
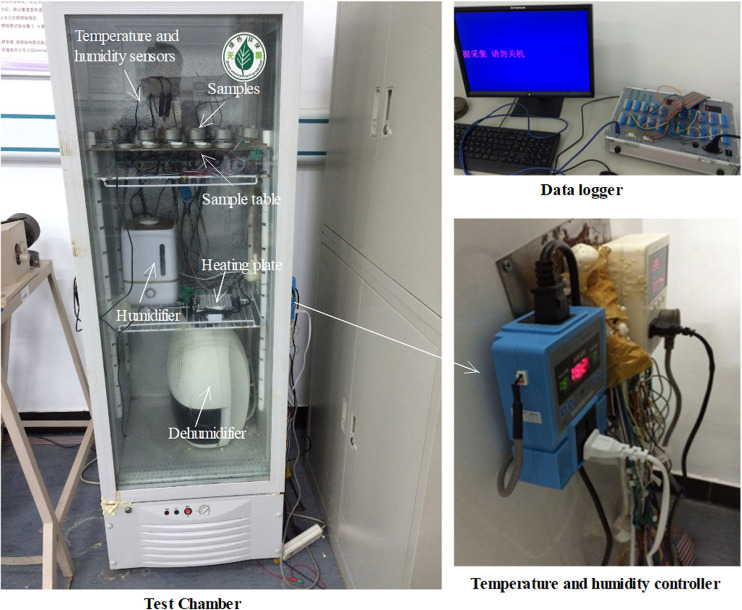
Hygroscopic experimental device.

To analyze the hygroscopic behavior of the rock under different temperature and relative humidity conditions, a custom-built constant temperature and humidity chamber was used for experiments (Fig 2). This chamber is equipped with temperature and humidity controllers that can adjust the internal environment’s temperature and humidity. The temperature control precision is ± 0.5°C, and the humidity control precision is ± 3%RH. Additionally, through temperature and humidity sensors and a data acquisition system, the system can obtain and record real-time changes in temperature and humidity throughout the experiments, with a data collection frequency of once per second.

In consideration of the actual construction environment during underground excavation, the experiments selected a temperature range typical of ambient conditions, from 5°C to 30°C. Specifically, four temperature environments were established: 5°C, 10°C, 20°C, and 30°C. Additionally, four relative humidity environments were set at 40% RH, 60% RH, 80% RH, and 100% RH to systematically study the hygroscopic behavior under various temperature and humidity conditions.

Each experimental and control group consisted of three samples, which were tested simultaneously within the same temperature and humidity environment. For investigating the effect of relative humidity on the rock’s hygroscopic behavior, the temperature was set to 20°C. Conversely, to examine the influence of temperature on hygroscopic behavior, the relative humidity was fixed at 100% RH in the experimental environment.

#### 2.2.2 . Experimental procedures.

First, place the dried rock samples (experimental groups) and quartz samples (control groups) into the temperature and humidity chamber for experimentation. At regular intervals (approximately every 10 hours), take the samples of both groups out of the chamber for weighing using an electronic balance with a measurement precision of ± 0.001 g. By calculating the change in sample mass, the water content at each time point can be easily determined using the following formula:


$$w=\frac{m_{1}-m_{0}}{m_{0}}\times 100\%$$ (1)

where *w* represents the water content after moisture absorption, *m*_1_ is the mass of the sample after moisture absorption, and *m*_0_ is the mass of the dried sample.

When the average mass or water content of the three samples in each group shows no significant fluctuations over three consecutive measurements, it indicates that the water content in the samples has reached a dynamic equilibrium under the corresponding environmental conditions. This means that the rate of moisture absorption from the air is equal to the rate of moisture loss from the samples. At this point, the hygroscopic experiment for the samples under the specified temperature or humidity conditions is considered complete.

### 2.3. Mechanical experiments

This study conducted mechanical experiments on reconstituted samples of the high clay-content shale to investigate their mechanical properties. To simulate the diagenesis process, an appropriate amount of water was added to rock particles before reconstitution, softening the clay particles and enhancing their plasticity and viscosity. Due to the water retention properties of clay minerals, some water remained in the reconstituted samples, resulting in samples with relatively high-water content. To obtain samples with varying water content, the reconstituted samples were placed in a drying oven for controlled water content.

**Fig 3:**
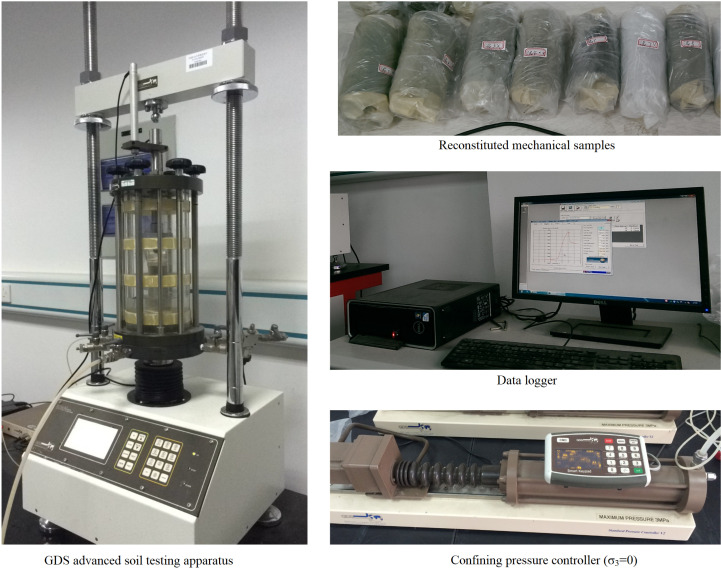
Mechanical testing apparatus and samples.

The high clay-content shale is a low-strength material, and given the limitations of rigid testing machines (such as MTS816) in providing accurate data, this study employed a GDS triaxial testing system to conduct uniaxial compression experiments on standard reconstituted shale samples with different water contents. The GDS system has a maximum axial load capacity of 50 kN, making it well-suited for mechanical testing of low-strength materials like high clay-content shale, ensuring precise and reliable results. The experiments followed a displacement-controlled loading mode with a loading rate of 1 mm/min to ensure the accuracy and comparability of the experimental data (Fig 3).

## 3. Results and discussion

### 3.1. Influence of humidity on water content

**Fig 4:**
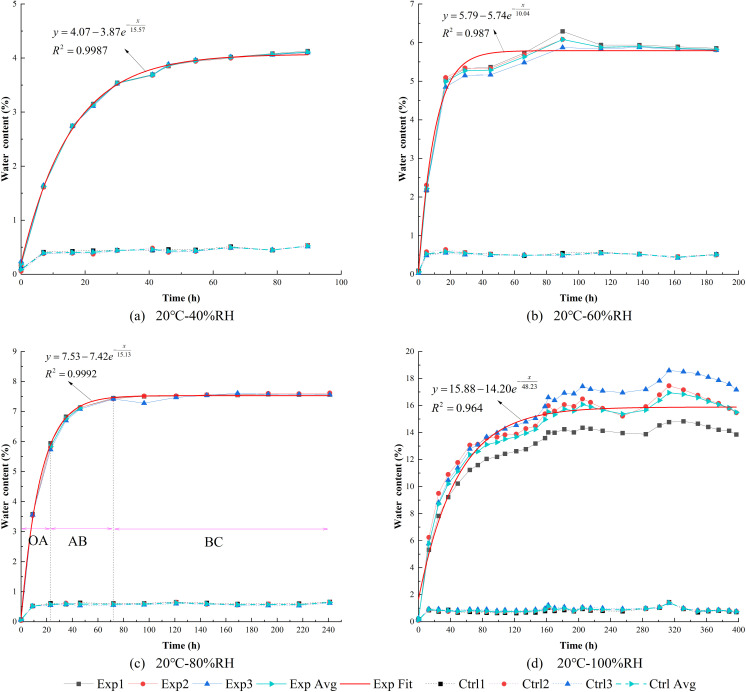
Water contents over time under different humidity.

**Fig 5:**
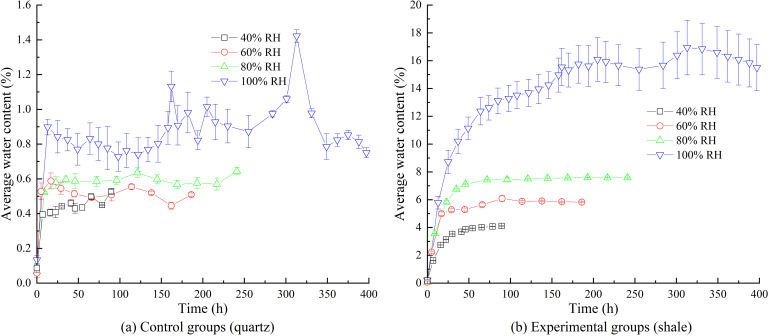
Experimental deviations of water contents over time under different humidity.

Under constant temperature conditions at 20°C, the variations in water contents over time for the control groups (quartz sand) and the experimental groups (high clay-content shale) at relative humidity levels of 40%RH, 60%RH, 80%RH, and 100%RH are shown in Fig 4. Each subplot in Fig 4 illustrates the time-dependent water content behavior for both the control and experimental groups under identical temperature and humidity conditions. The water content variations for individual samples from the control and experimental groups under different humidity conditions are presented in Fig 5.

#### 3.1.1. EWC and deviations in the control groups.

As shown in Fig 4, the water contents of the control groups during the experiment were significantly lower than those of the experimental groups. According to Fig 5(a) and S6, the EWC of the control groups was approximately 0.45% at 40%RH, increased to about 0.52% at 60%RH, reached approximately 0.59% at 80%RH, and reached 0.88% at 100%RH. This indicates that the EWC of non-clay rocks increases with higher relative humidity. Since quartz does not have hygroscopic properties and cannot form bound water with moisture, the water mainly exists as free water in structural joints and pores.

When the relative humidity was less than or equal to 80%RH, the deviations in water content among the three samples in the control groups were minimal during the experiment. However, when the relative humidity reached 100%RH, the deviations in water content among the samples increased significantly, indicating that high humidity is a critical factor affecting the variability of water content in non-clay rocks.

Under high-humidity conditions (100%RH), significant deviations in water content were observed among the control group samples during the early and middle stages of the experiment (approximately within 270 hours). This was primarily due to variations in the adsorption capacity of the particle surfaces. Goss [38] reported that the adsorption behavior of quartz sand for volatile organic compounds is significantly influenced by the number of active sites and chemical properties of the particle surfaces, particularly the distribution of surface hydroxyl groups and the heterogeneity of microstructures. This finding aligns with the initial deviations in water content observed among samples in the high-humidity conditions in this study. Over time, surface adsorption gradually reached saturation, and water diffusion within the particles began to dominate. Since the deviations in internal particle structures have a smaller impact on adsorption capacity, the initial deviations in adsorption gradually weakened. Ultimately, the water content of the three samples converged, and the deviations in water content diminished over time.

Additionally, the initial water content data collected from the control groups were nearly equal to the EWC, and subsequent fluctuations were minimal. This indicates that for non-clay rocks, the time required to reach EWC is very short.

#### 3.1.2. EWC and deviations in the experimental groups.

As shown in Figs 4 and 5(b), the EWC of high clay-content shale increases significantly with rising humidity. At 40%RH, the EWC is approximately 4.11%; it increases to about 5.80% at 60%RH, 7.58% at 80%RH, and reaches 15.83% at 100%RH.

**Fig 6:**
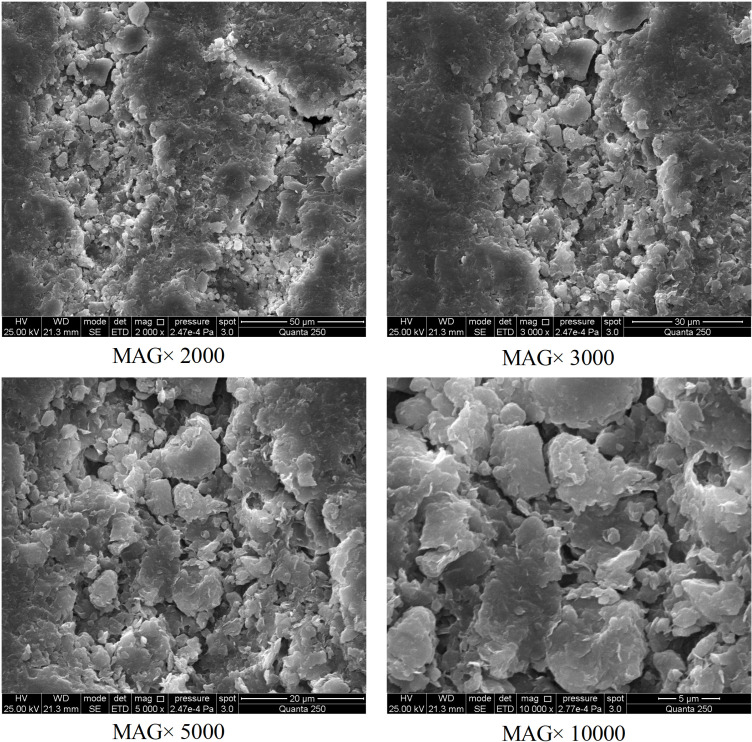
Microscopic structure of high clay-content shale (SEM scan results).

Similar to the control groups, when the relative humidity is less than or equal to 80%RH, the deviations in water content among the samples in the experimental groups are minimal, showing high consistency. However, when the relative humidity rises to 100%RH, the variation trend in the experimental group samples differs significantly from that of the control group. During the initial stage of moisture absorption, the deviations in water content among the samples are small. As absorption time extends, the deviations gradually increase until the EWC is reached. This phenomenon is primarily attributed to the randomly and unevenly distributed micropores and microcracks within the high clay-content shale (as shown in Fig 6). These microstructures significantly influence moisture absorption behavior under high-humidity conditions. Studies by Feng et al. [39], Sang et al. [40], Liu et al. [41], and Shen et al. [42] indicate that under high-humidity conditions, the type, shape, and structure of pores and cracks play a critical role in forming deviations in moisture absorption, with capillary condensation effects further amplifying these deviations.

However, once the samples reach EWC, these deviations no longer change significantly. This may be due to the irreversible adsorption properties of high clay-content shale, meaning that adsorbed water cannot be fully desorbed under 20°C and 100%RH conditions. Ma et al. [43] reported that the water adsorption-desorption curve of shale exhibits significant hysteresis effects. In particular, in small pores, capillary pressure makes desorption of water considerably more difficult.

Additionally, in the high-humidity environment of 100%RH, the average water content at the first measurement (13 hours) is approximately 5.79%. Although this is lower than the EWC of 7.58% under 80%RH conditions, the variation among the samples is significantly greater than under low-humidity conditions. This suggests that the deviations in water content among the experimental group samples are not directly related to the post-absorption water content but are determined by the humidity environment.

Furthermore, water content has a significant impact on the expansion coefficient of shale [44]. This indicates that in practical engineering, high clay-content shale, due to the large exposed area of the free surface, is prone to moisture absorption deviations. These deviations result in uneven water distribution, which in turn causes non-uniform expansion and cracking of the rock mass. Therefore, reducing environmental humidity is an effective measure to mitigate these effects.

#### 3.1.3. Hygroscopic parameters quantification based on humidity.

To quantitatively analyze the effects of humidity on the hygroscopic intensity, characteristic time, and EWC of high clay-content shale, an exponential function was used to fit the average water content curves, as shown by the red curves in Fig 4. The corresponding fitting parameters are detailed in Table 2. The general form of the exponential fitting function is:

**Table 2:** Hygroscopic parameters of the fitted moisture absorption curves of the experiment samples under different relative humidity.

Number	Humidity environment	*R* ^2^	*y*_0_ (%)	*n* (%)	*t* (h)
Fig 4(a)	40%RH	0.998	4.07	3.87	15.57
Fig 4(b)	60%RH	0.987	5.79	5.74	10.04
Fig 4(c)	80%RH	0.999	7.53	7.42	15.13
Fig 4(d)	100%RH	0.964	15.88	14.20	48.23


$$y=y_{0}-ne^{-\frac{x}{t}}$$ (2)

where *x* represents the hygroscopic time, *y* represents the water content, *y*_0_ represents EWC, *t* represents the characteristic time parameter indicating the time required to reach EWC, and *n* represents the initial hygroscopic intensity.

The coefficient of determination *R*^2^ for the fitted curve averaged 0.987, indicating that the exponential function fitting is well-suited for this analysis. The moisture absorption process can be divided into three stages: the rapid hygroscopic stage (segment OA), the decelerated hygroscopic stage (segment AB), and the stable stage (segment BC), as shown in Fig 4(c). This trend is consistent with the experimental results of Erguler et al. [14] and Garcia-Fernandez et al. [15].

During the rapid hygroscopic stage (segment OA), the hygroscopic rate increased nearly linearly. Within approximately 20 hours, the water content quickly increased to 6%. This suggests that, in rock mass engineering, it is crucial to quickly seal the surrounding rock to prevent rapid moisture absorption from the air after excavation, which would increase the water content, reduce the rock strength, and ultimately affect structural stability. The decelerated hygroscopic stage (segment AB) is characterized by a slowing hygroscopic rate over time until the stable stage (segment BC) is reached, where the water content reaches equilibrium and remains constant within a specific range.

**Fig 7:**
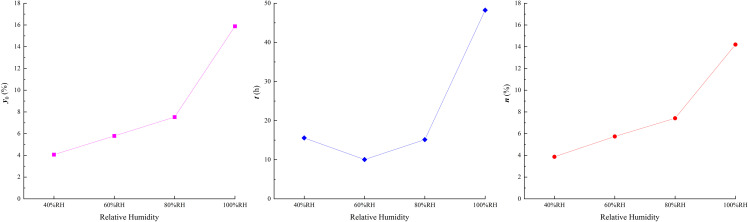
Fitting parameters of moisture absorption vs. relative humidity.

Under varying humidity conditions, the relationships between the fitting parameters and relative humidity are shown in Fig 7.

As observed in Fig 7, the fitting parameters generally increase with increasing humidity. The EWC (*y*_0_) increases linearly within the 40%–80%RH range, with a relatively slow growth rate, but rises significantly in the 80%–100%RH range. This trend is consistent with the findings of Trugilho et al. [45], Barker et al. [46], and Pixton et al. [47], who reported that the EWC of materials exhibits a near-linear relationship with relative humidity under low-humidity conditions. These results suggest that reducing relative humidity is an effective way to lower the EWC after moisture absorption.

The initial hygroscopic intensity (*n*) exhibits a similar trend to *y*_0_, indicating that higher humidity leads to greater initial hygroscopic intensity and a faster increase in water content. This is further supported by the slope of water content within 20 hours, as shown in Fig 5(b).

The variation in characteristic time (*t*) with humidity shows that in low-humidity conditions (40%–80%RH), *t* remains relatively stable, indicating that the time required to reach EWC is similar within this range. For example, high clay-content shale under 40%–80%RH conditions reaches EWC within approximately 50 hours, as shown in Fig 5(b). However, under high-humidity conditions, *t* increases significantly, requiring about 200 hours to reach EWC.

These findings demonstrate that reducing humidity effectively lowers the EWC, initial hygroscopic intensity, and characteristic time of high clay-content shale after moisture absorption.

### 3.2. Influence of temperature on water content

**Fig 8:**
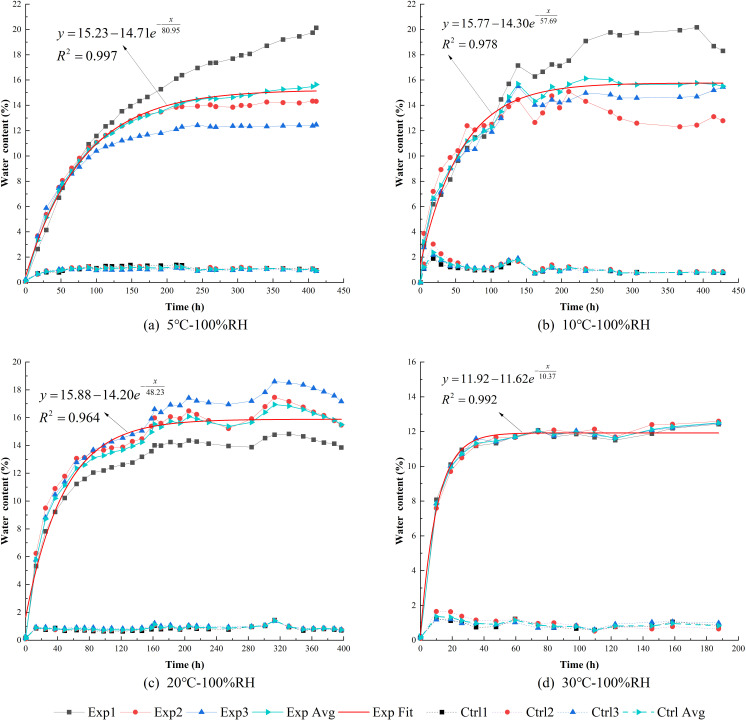
Water contents over time under different temperature.

**Fig 9:**
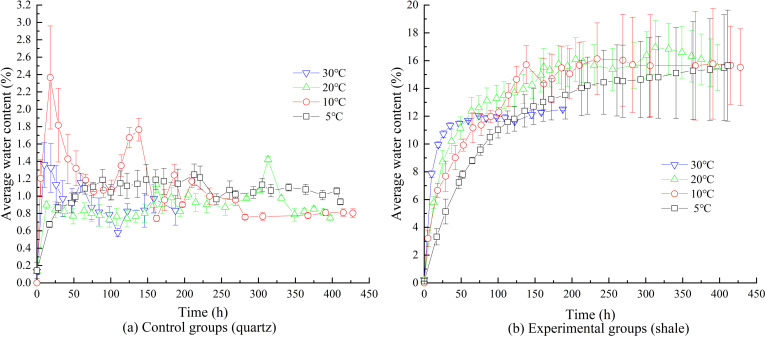
Experimental deviations of water contents over time under different temperature.

Under a constant relative humidity of 100%RH, the temperature was respectively set at 5°C, 10°C, 20°C, and 30°C. The variations in water contents over time for the control groups and experimental groups are shown in Fig 8. Each subplot in Fig 8 illustrates the time-dependent trends of the water contents under identical temperature and humidity conditions for both groups. Detailed variations in water contents over time for individual samples under different temperature conditions are presented in Fig 9.

#### 3.2.1. EWC and deviations in the control groups.

Under different temperature conditions, the water contents of the control groups remained significantly lower than those of the experimental groups, with EWC being approximately 1%. As the temperature increased, the EWC decreased slightly but remained close to 1%. Under conditions of 10°C and 20°C, the deviations in water content among the three samples in the control groups were more pronounced during the early stages of the experiment but gradually diminished over time. For the analysis of the time-dependent variation in water content deviations under high-humidity conditions, refer to Section 3.1.1.

#### 3.2.2. EWC and deviations in the experimental groups.

As shown in Figs 8 and 9(b), under relatively low-temperature conditions (5°C–20°C), the average EWC of the three samples is approximately 15.6%. However, at 30°C, the EWC decreases to 12%, indicating that a relatively high temperature (30°C) is more effective in reducing EWC.

Furthermore, the lower the temperature, the greater the deviations in water content among the samples after reaching equilibrium. Specifically, under relatively low-temperature conditions (5°C–20°C), the deviations in water contents among the samples increase during the early and middle stages of moisture absorption. At 5°C, these deviations continue to grow even after the EWC is reached, eventually exceeding the deviations observed at 10°C. During the later stages of moisture absorption at 10°C, the deviations among samples slightly decrease, but the reduced values remain higher than those at 20°C. At 20°C, as discussed in Section 3.1.2, the deviations in EWC remain constant. When the temperature reaches 30°C, the deviations throughout the entire moisture absorption process are minimal, suggesting that under high-humidity conditions, a higher temperature (30°C) effectively eliminates the moisture absorption deviations caused by high humidity.

This phenomenon may be attributed to the significantly enhanced evaporation capacity of water, especially free water, within high clay-content shale at higher temperatures. This enhanced evaporation reduces the impact of microstructural differences, such as variations in micropores and microcracks, within the rock. Conversely, at lower temperatures, the evaporation capacity of water decreases, allowing free water to accumulate in randomly distributed and unevenly sized micropores and microcracks. The size of these pores and cracks determines the storage capacity of free water, amplifying the deviations as the temperature decreases.

Therefore, in practical engineering applications, in addition to reducing humidity as discussed in Section 3.1.2, increasing the temperature can also be an effective strategy to mitigate moisture absorption deviations and reduce uneven water distribution within the rock mass.

#### 3.2.3. Hygroscopic parameters quantification based on temperature.

**Fig 10:**
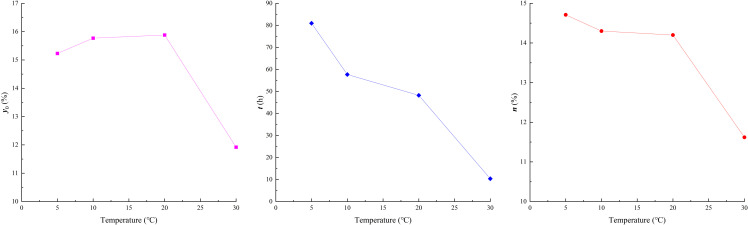
Fitting parameters of moisture absorption vs. temperature.

To quantitatively analyze the effects of humidity on the hygroscopic intensity, characteristic time, and EWC of high clay-content shale, equation (2) was used to fit the average water content curves under different temperature conditions (as shown by the red curves in Fig 8). The corresponding fitting parameters are listed in Table 3, and their relationships with temperature are shown in Fig 10.

**Table 3:** Relevant parameters of the fitted moisture absorption curves of the experiment samples under different temperature.

Number	Temperature environment	*R* ^2^	*y*_0_ (%)	*n* (%)	*t* (h)
Fig 8(a)	5°C	0.997	15.23	14.71	80.95
Fig 8(b)	10°C	0.978	15.77	14.30	57.69
Fig 8(c)	20°C	0.964	15.88	14.20	48.23
Fig 8(d)	30°C	0.992	11.92	11.62	10.77

Under different temperature, the coefficient of determination (R²) for the moisture absorption fitting curves of each group in the experimental set had a maximum value of 0.997, a minimum value of 0.964, and an average of 0.982. This indicates that the fitting function is highly reliable for describing the hygroscopic effects under various temperature.

As shown in Fig 10, the EWC (*y*_0_) slightly increases within the low-temperature range (5°C–20°C), rising from 15.23% (5°C) to 15.88% (20°C). This may be due to the increased kinetic energy of water molecules in the air as the temperature rises, which enhances the probability of contact between water vapor and clay minerals, leading to a slight increase in EWC. Martin [48] also reported that elevated temperatures enhance the diffusion and mobility of water molecules, altering the adsorption properties of clay surfaces and increasing the likelihood of water-vapor interaction. However, under higher temperature conditions (30°C), the EWC (*y*_0_) decreases to 11.92%. This is attributed to the enhanced evaporation capacity of water in high clay-content shale as the temperature increases, making it difficult for free water to accumulate in micropores and microcracks within the microstructure.

The characteristic time (*t*) decreases gradually with increasing temperature, showing an approximately linear relationship. This indicates that higher temperatures significantly reduce the time required for high clay-content shale to reach EWC.

The initial hygroscopic intensity (*n*) also decreases with increasing temperature. In the low-temperature range (5°C–20°C), the reduction is relatively small, from 14.71% (5°C) to 14.20% (20°C). However, at a higher temperature (30°C), the reduction is more pronounced, decreasing to 11.62%. The reduction in initial hygroscopic intensity also contributes to the decrease in EWC.

In summary, higher environmental temperatures, particularly 30°C, effectively reduce the EWC, initial hygroscopic intensity, and characteristic time of high clay-content shale.

### 3.3. Comparison of temperature and humidity effects on water content

Based on the fitting results, the effects of temperature and humidity on the water content of rock samples differ significantly. By controlling the environmental humidity, the water content can be reduced from 15.88% (at 100% RH) to 4.07% (at 40% RH); however, by adjusting the temperature, the water content only decreases from 15.88% (at 5°C-20°C) to 11.92% (at 30°C). This indicates that reducing the relative humidity has a more pronounced effect on rock water content than temperature adjustments. It also highlights that humidity is the primary factor influencing the EWC of high clay-content shale, while temperature plays a supplementary role.

For underground engineering, where technical and equipment conditions allow, adjusting the working environment’s humidity is more effective than regulating temperature in controlling rock water content.

### 3.4. Influence of water content on the mechanical properties

**Fig 11:**
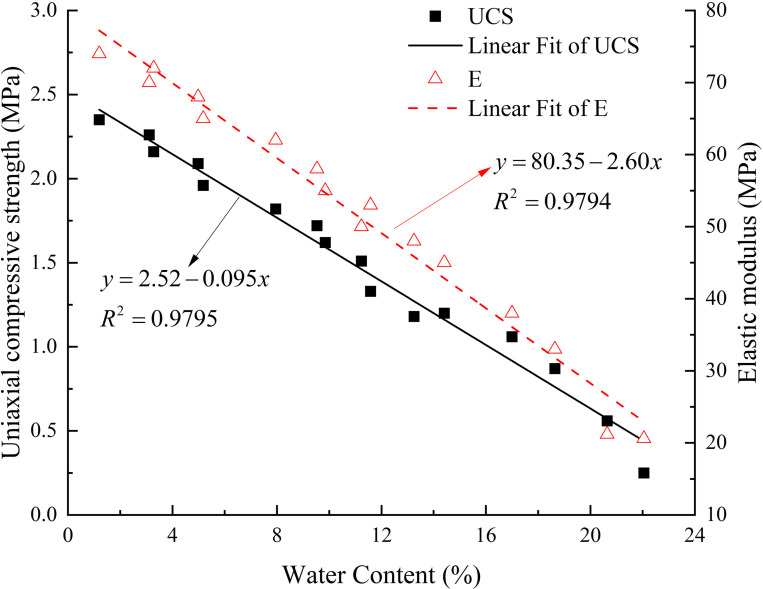
Relationship between UCS, E, and water content.

Fig 11 shows that the UCS and E (S7) decrease approximately linearly as water content increases, with a high coefficient of determination in the fit line (R² ≈  0.979). According to the fitting results, the UCS and E of dry rock are 2.52 MPa and 80.35 MPa, respectively. Based on the hygroscopic fitting results (Fig 10 and Table 3), in high-humidity conditions, the average water content of rocks after moisture absorption is 14.7%, with corresponding UCS and E values of 1.12 MPa and 42.13 MPa. This indicates that after moisture absorption, the UCS and E of high clay-content shale decrease by approximately 55.56% and 47.57%, respectively.

The linear decrease in UCS and E with increasing water content can be attributed to the following mechanisms: First, water molecules adsorbed on the clay surface form a lubrication layer, weakening the cohesive and frictional forces between mineral particles, thereby reducing the overall mechanical properties [49]. Second, the water in high clay-content shale generates pore water pressure during loading, which affects the stress distribution within the rock, further reducing its compressive strength [50].

Under low-humidity condition, the EWC of the rock after moisture absorption is 4.07% (Fig 4 and Table 2), with corresponding UCS and E values of 2.13 MPa and 77.57 MPa. Compared to the dry state, the UCS and E decrease by only about 15.48% and 3.46%, respectively. This smaller decrease is because, under low-humidity conditions, water cannot deeply penetrate micropores and microcracks, better preserving the structural integrity of the rock.

In terms of the impact of temperature, when the temperature increases from 5°C to 30°C, the EWC of the rock after moisture absorption decreases from 15.23% to 11.92% (as shown in Table 3), and the corresponding UCS and E increase from 1.07 MPa and 40.75 MPa to 1.39 MPa and 49.36 MPa, respectively. This phenomenon can be attributed to the increased kinetic energy of water molecules at higher temperatures, which promotes water evaporation, reduces the EWC, and thereby lessens the weakening effect of water on the cohesive forces between particles, effectively mitigating the deterioration of mechanical properties [51].

The results indicate that adjusting environmental humidity and temperature can significantly reduce the weakening effects of moisture absorption on the mechanical properties of rock. Notably, reducing humidity is a more efficient strategy for optimizing the long-term mechanical performance of rock.

## 4. Conclusions

This study systematically investigated the hygroscopic behavior of high clay-content shale under varying temperature and humidity conditions, as well as its impact on mechanical properties, through moisture absorption and mechanical performance experiments. The main conclusions are as follows:

(1) The moisture absorption process of high clay-content shale exhibits an exponential growth pattern over time, consisting of rapid hygroscopic stage, decelerated hygroscopic stage, and stable stage. Under high relative humidity (100% RH), the rapid hygroscopic stage lasts approximately 50 hours, whereas under low relative humidity, it lasts around 20 hours. Humidity is the primary factor determining EWC, while the effect of temperature is relatively minor.
(2) High humidity and relatively low temperatures can cause deviations in the water content of the specimens after moisture absorption. Under low humidity conditions, the water content deviations of high clay-content shale after moisture absorption are minimal. However, under high humidity conditions, the deviations become significant, particularly at relatively low temperatures. During the moisture absorption process, the deviations gradually increase over time until the equilibrium water content state is reached. At the equilibrium state, the deviations continue to increase at 5°C; as the temperature rises, the deviations begin to decrease (10°C) and eventually stabilizes (20°C). When the temperature reaches 30°C, the deviation throughout the entire moisture absorption process almost disappears.
(3) In high-humidity environments (100% RH), the EWC increases significantly, leading to reductions of approximately 50% in the UCS and E compared to dry samples. Conversely, lowering the humidity to 40% RH limits the EWC to around 4%, with corresponding decreases in the UCS and E of only 15.48% and 3.46%, respectively. Increasing the temperature from 5°C to 30°C moderately reduces EWC and mitigates mechanical degradation, but its impact is far less pronounced than that of humidity control. The UCS and E of high clay-content shale decrease approximately linearly with increasing water content. These mechanical properties are significantly more sensitive to changes in relative humidity than to temperature.
(4) Controlling environmental humidity is crucial for reducing the water content and mitigating the weakening of mechanical properties in high clay-content shale. Although temperature adjustments can serve as a supplementary measure, maintaining low-humidity conditions is the most effective strategy for enhancing the stability of underground structures.

## Supporting information

S1 Fig XRD diffraction pattern of the shale. (DOCX)

S2 Fig Weathering the shale.
(DOCX)

S3 Fig Argillitization of the shale.
(DOCX)

S4 Fig Preparation of hygroscopic samples.
(DOCX)

S5 Fig The reconstitution process of high clay-content shale. (DOCX)

S6 Table Measured results of moisture absorption for experiment groups and control groups.
(DOCX)

S7 Table Water content, uniaxial compressive strength, and elastic modulus of reconstructed shale.
(DOCX)
