# Supplementary figures and images for: Hygroscopic effect of high clay-content shale under temperature and humidity conditions and its impact on mechanical properties
Source: PLoS One. 2025 Mar 7;20(3):e0319672. doi: 10.1371/journal.pone.0319672 (PMC11888144; doi:10.1371/journal.pone.0319672)

**S1 Fig. XRD diffraction pattern of the shale**

**
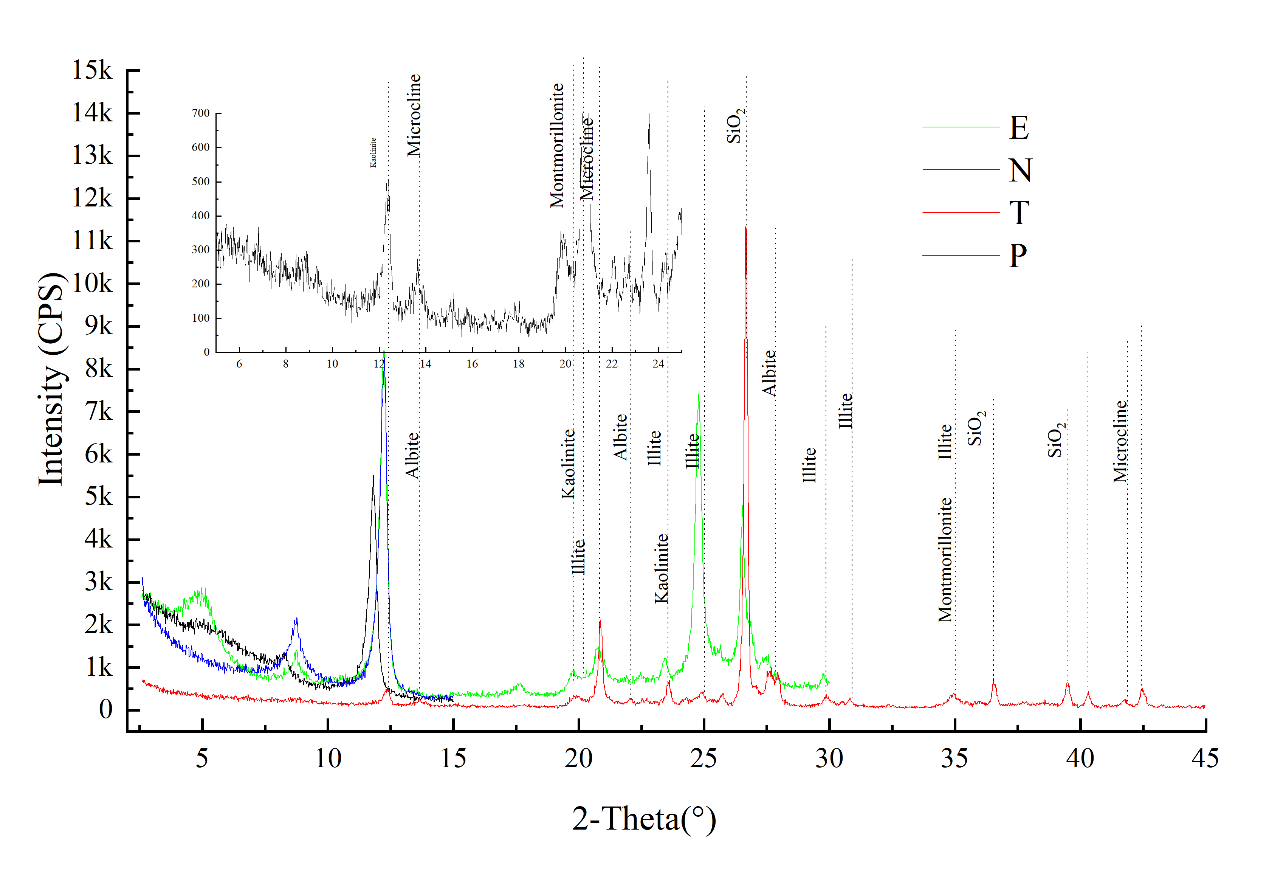
**

Supplement: S1 Fig — (DOCX) [file pone.0319672.s001.docx]

**S2 Fig. Weathering of the shale**


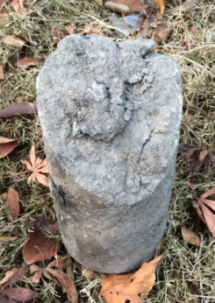

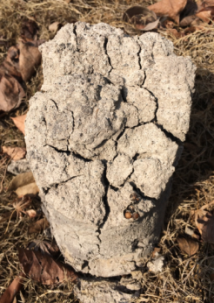

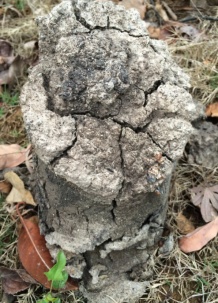


(a) 0 d (b) 60 d (c) 120 d

Supplement: S2 Fig — (DOCX) [file pone.0319672.s002.docx]

**S3 Fig. Argillitization of the shale**


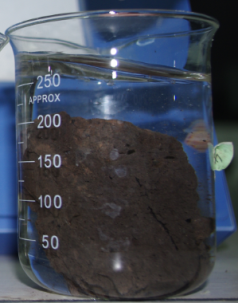

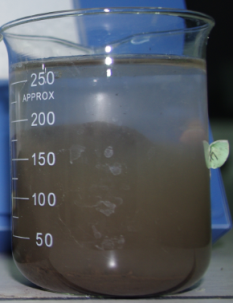


(a) 0 min (b) 3 min


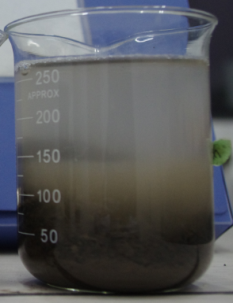

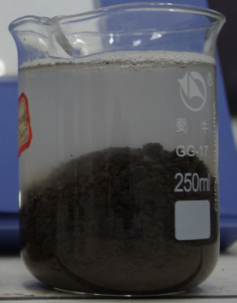


(c) 7 min (d) 10 min

Supplement: S3 Fig — (DOCX) [file pone.0319672.s003.docx]

**S4 Fig.** **Preparation of hygroscopic samples**


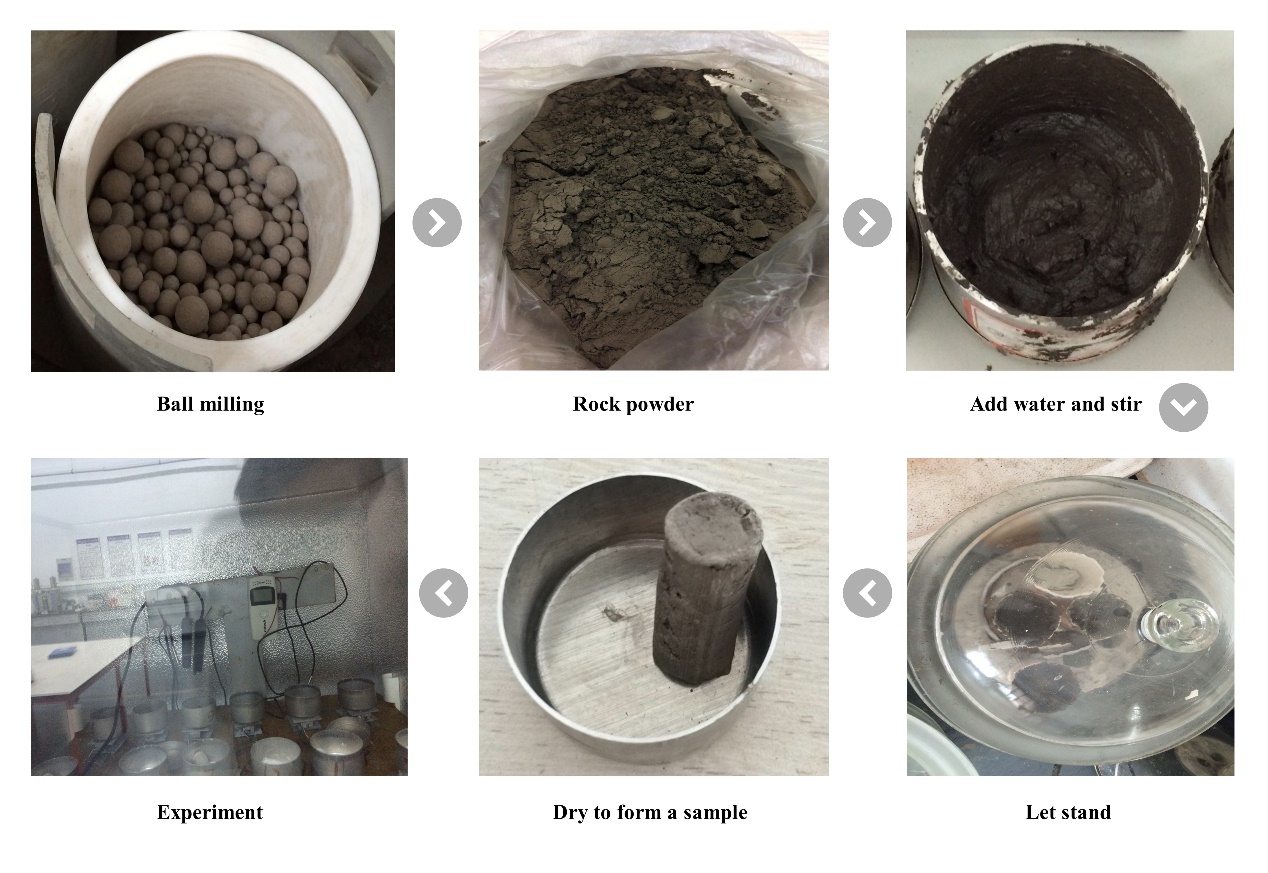

Supplement: S4 Fig — (DOCX) [file pone.0319672.s004.docx]
