# Supplementary material for: Hygroscopic effect of high clay-content shale under temperature and humidity conditions and its impact on mechanical properties
Source: PLoS One. 2025 Mar 7;20(3):e0319672. doi: 10.1371/journal.pone.0319672 (PMC11888144; doi:10.1371/journal.pone.0319672)
Supplement: S5 Fig — (DOCX) [file pone.0319672.s005.docx]

**S5** **Preparation of reconstituted mechanical samples**

**Fig. Reconstruction platform**


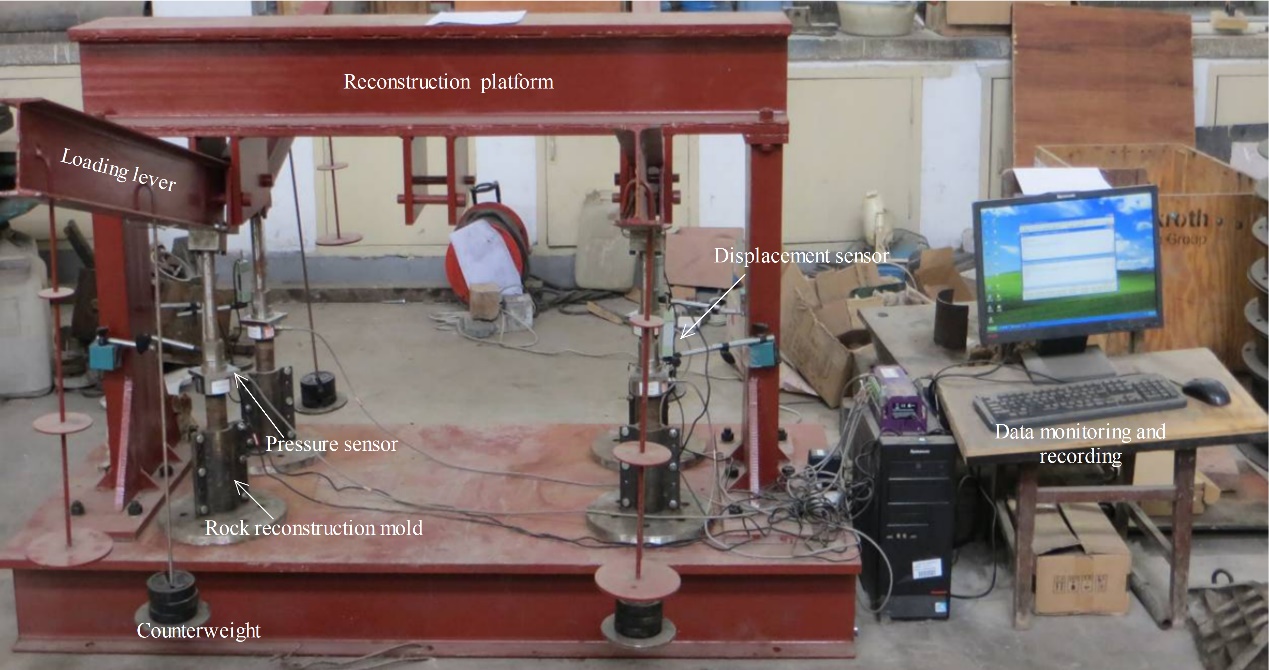


**Materials and Mineral Composition**

The reconstituted samples were prepared using crushed fragments of the original high clay-content shale, ensuring that the mineral composition remained consistent with the natural rock. The mineral composition of the original rock is detailed in **Table 1 of the manuscript**.

**Compaction Process**

The compaction process involved **stepwise loading** applied using a custom-designed reconstruction platform to simulate diagenesis. The applied loads are shown in the following **Table**, with four levels of loading:

**Tab. Application of stepwise reconstruction load**

| **Design reconstruction load**  **σ_z_ (MPa)** | **Stepwise loading (MPa)** | | | |
| --- | --- | --- | --- | --- |
|  | **1st level** | **2nd level** | **3rd level** | **4st level** |
| **7.5** | **1.25** | **2.5** | **5** | **7.5** |

- At each load level, axial displacement was monitored using a high-precision sensor. The load was maintained until the displacement remained stable for **24 hours**, indicating the sample had reached a consolidated state.
- This process was repeated for all four loading levels. Once the final load was applied and axial displacement remained stable for 24 hours, the reconstituted sample was deemed complete.

**Consolidation Conditions**

To ensure the reconstituted samples closely resembled natural diagenetic conditions:

1. **Water Addition:** A measured amount of water was added to the crushed rock to enhance plasticity and facilitate compaction. The amount of water was determined based on the initial moisture content of the crushed rock, ensuring a plastic state suitable for compaction.
2. **Drainage Consolidation:** A steel permeable block was placed at the bottom of the reconstruction mold to allow for water drainage during the compaction process.

**Limitations and Purpose**

Due to the difficulty of obtaining intact original rock samples for direct mechanical testing, the reconstituted samples were prepared as a substitute. These samples were used to investigate the mechanical behavior of high clay-content shale under varying water content conditions. The reconstruction process ensures that the samples meet the experimental requirements for studying the relationship between water content and mechanical properties.
