# Supplementary material for: Hygroscopic effect of high clay-content shale under temperature and humidity conditions and its impact on mechanical properties
Source: PLoS One. 2025 Mar 7;20(3):e0319672. doi: 10.1371/journal.pone.0319672 (PMC11888144; doi:10.1371/journal.pone.0319672)
Supplement: S6 Table — (DOCX) [file pone.0319672.s006.docx]

**S6 Tab.** **Measured results of moisture absorption for experiment groups and control groups**

**Effect of humidity on the water content of high clay-content shale**

**20℃-40%RH**

| **Absorption time (hours)** | **Water content of the experiment group (Shale) (%)** | | | | **Water content of the control group (quartz) (%)** | | | |
| --- | --- | --- | --- | --- | --- | --- | --- | --- |
|  | **Sample 1** | **Sample 2** | **Sample 3** | **Average** | **Sample 1** | **Sample 2** | **Sample 3** | **Average** |
| 0 | 0.16677 | 0.16437 | 0.23717 | 0.18943 | 0.09561 | 0.0569 | 0.10749 | 0.08667 |
| 7 | 1.6183 | 1.61064 | 1.64442 | 1.62445 | 0.41132 | 0.38253 | 0.39028 | 0.39471 |
| 16 | 2.74727 | 2.73552 | 2.73894 | 2.74057 | 0.42849 | 0.39018 | 0.39874 | 0.4058 |
| 22.5 | 3.15102 | 3.13176 | 3.11127 | 3.13135 | 0.43911 | 0.37392 | 0.41261 | 0.40855 |
| 30 | 3.5438 | 3.528 | 3.52695 | 3.53292 | 0.4462 | 0.43895 | 0.44035 | 0.44183 |
| 41 | 3.69521 | 3.68062 | 3.69272 | 3.68952 | 0.4462 | 0.48365 | 0.45075 | 0.4602 |
| 46 | 3.8532 | 3.8626 | 3.88653 | 3.86744 | 0.46036 | 0.40643 | 0.42302 | 0.42994 |
| 54.5 | 3.95633 | 3.93891 | 3.96304 | 3.95276 | 0.45328 | 0.42269 | 0.42995 | 0.43531 |
| 65.5 | 4.01996 | 3.99761 | 4.02170 | 4.01309 | 0.51348 | 0.49178 | 0.4889 | 0.49805 |
| 78.5 | 4.08454 | 4.06785 | 4.06196 | 4.07145 | 0.4462 | 0.45114 | 0.45075 | 0.44936 |
| 89.5 | 4.12961 | 4.09819 | 4.10497 | 4.11092 | 0.53473 | 0.53243 | 0.51317 | 0.52677 |

**20℃-60%RH**

| **Absorption time (hours)** | **Water content of the experiment group (Shale) (%)** | | | | **Water content of the control group (quartz) (%)** | | | |
| --- | --- | --- | --- | --- | --- | --- | --- | --- |
|  | **Sample 1** | **Sample 2** | **Sample 3** | **Average** | **Sample 1** | **Sample 2** | **Sample 3** | **Average** |
| 0 | 0.09753 | 0.09425 | 0.02829 | 0.07336 | 0.08232 | 0.06182 | 0.02918 | 0.05777 |
| 5 | 2.17667 | 2.30909 | 2.18224 | 2.22267 | 0.51349 | 0.58187 | 0.48874 | 0.52804 |
| 17 | 5.02497 | 5.09584 | 4.85144 | 4.99075 | 0.56837 | 0.64006 | 0.5544 | 0.58761 |
| 29 | 5.32864 | 5.34217 | 5.14847 | 5.27309 | 0.56445 | 0.56369 | 0.50698 | 0.54504 |
| 45 | 5.36632 | 5.32932 | 5.16867 | 5.28811 | 0.52525 | 0.52369 | 0.49239 | 0.51378 |
| 66 | 5.73206 | 5.6849 | 5.48389 | 5.63361 | 0.47822 | 0.50187 | 0.49604 | 0.49204 |
| 90 | 6.29285 | 6.08331 | 5.87386 | 6.08334 | 0.54877 | 0.49459 | 0.48145 | 0.50827 |
| 114 | 5.9382 | 5.87125 | 5.83547 | 5.88164 | 0.56837 | 0.55642 | 0.53981 | 0.55486 |
| 138 | 5.93598 | 5.89481 | 5.8779 | 5.9029 | 0.52525 | 0.52369 | 0.51063 | 0.51986 |
| 162 | 5.89165 | 5.84126 | 5.82537 | 5.85276 | 0.4547 | 0.45823 | 0.42309 | 0.44534 |
| 186 | 5.85619 | 5.81342 | 5.80516 | 5.82492 | 0.51741 | 0.49823 | 0.51063 | 0.50876 |

**20℃-80%RH**

| **Absorption time (hours)** | **Water content of the experiment group (Shale) (%)** | | | | **Water content of the control group (quartz) (%)** | | | |
| --- | --- | --- | --- | --- | --- | --- | --- | --- |
|  | **Sample 1** | **Sample 2** | **Sample 3** | **Average** | **Sample 1** | **Sample 2** | **Sample 3** | **Average** |
| 0 | 0.0761 | 0.04038 | 0.05771 | 0.05806 | 0.06051 | 0.05654 | 0.08271 | 0.06659 |
| 9 | 3.58524 | 3.54093 | 3.56087 | 3.56235 | 0.52733 | 0.5188 | 0.51944 | 0.52186 |
| 23 | 5.9465 | 5.81407 | 5.73458 | 5.83172 | 0.61666 | 0.56535 | 0.55253 | 0.57818 |
| 35 | 6.82801 | 6.75885 | 6.70068 | 6.76252 | 0.58784 | 0.61856 | 0.58231 | 0.59624 |
| 46 | 7.13454 | 7.13838 | 7.08327 | 7.11873 | 0.63107 | 0.58863 | 0.5426 | 0.58744 |
| 72 | 7.4474 | 7.42303 | 7.41884 | 7.42975 | 0.61378 | 0.58863 | 0.55584 | 0.58608 |
| 96 | 7.50447 | 7.51791 | 7.27777 | 7.43339 | 0.6109 | 0.59529 | 0.56576 | 0.59065 |
| 121 | 7.51293 | 7.51993 | 7.47013 | 7.501 | 0.65412 | 0.6485 | 0.60216 | 0.63492 |
| 145 | 7.55309 | 7.55627 | 7.54067 | 7.55001 | 0.62819 | 0.58198 | 0.58231 | 0.59749 |
| 169 | 7.54887 | 7.55627 | 7.60906 | 7.5714 | 0.59361 | 0.5587 | 0.54922 | 0.56718 |
| 193 | 7.57635 | 7.6027 | 7.57273 | 7.58393 | 0.59361 | 0.59529 | 0.53929 | 0.57606 |
| 217 | 7.56578 | 7.59059 | 7.56845 | 7.57494 | 0.60513 | 0.56868 | 0.53599 | 0.56993 |
| 241 | 7.56578 | 7.61885 | 7.56204 | 7.58222 | 0.66277 | 0.6485 | 0.62201 | 0.64442 |

**20℃-100%RH**

| **Absorption time (hours)** | **Water content of the experiment group (Shale) (%)** | | | | **Water content of the control group (quartz) (%)** | | | |
| --- | --- | --- | --- | --- | --- | --- | --- | --- |
|  | **Sample 1** | **Sample 2** | **Sample 3** | **Average** | **Sample 1** | **Sample 2** | **Sample 3** | **Average** |
| 0 | 0.18885 | 0.18716 | 0.26948 | 0.21517 | 0.14492 | 0.10183 | 0.1517 | 0.13282 |
| 13 | 5.32814 | 6.24165 | 5.78754 | 5.78578 | 0.85651 | 0.89117 | 0.94614 | 0.89794 |
| 25 | 7.82861 | 9.49769 | 8.82285 | 8.71638 | 0.75947 | 0.82451 | 0.94266 | 0.84221 |
| 37 | 9.22472 | 10.90172 | 10.46758 | 10.19801 | 0.88605 | 0.75785 | 0.83135 | 0.82508 |
| 49 | 10.22818 | 11.78948 | 11.38706 | 11.13491 | 0.68352 | 0.76135 | 0.86614 | 0.77034 |
| 64 | 11.23709 | 13.07332 | 12.78383 | 12.36475 | 0.73837 | 0.83854 | 0.91831 | 0.83174 |
| 74 | 11.59157 | 13.13069 | 13.09584 | 12.60603 | 0.71728 | 0.7789 | 0.9044 | 0.80019 |
| 85 | 12.04967 | 13.59779 | 13.68058 | 13.10935 | 0.66664 | 0.75083 | 0.91136 | 0.77628 |
| 98 | 12.20237 | 13.66334 | 13.97399 | 13.2799 | 0.64977 | 0.71574 | 0.81744 | 0.72765 |
| 108 | 12.43142 | 13.8327 | 14.27773 | 13.51395 | 0.67086 | 0.75083 | 0.86614 | 0.76261 |
| 122 | 12.6223 | 13.8846 | 14.54221 | 13.68303 | 0.64555 | 0.73679 | 0.83831 | 0.74022 |
| 134 | 12.77227 | 14.29434 | 14.79842 | 13.95501 | 0.69196 | 0.78241 | 0.83135 | 0.76857 |
| 146 | 13.18674 | 14.47189 | 15.06083 | 14.23982 | 0.68774 | 0.821 | 0.89744 | 0.80206 |
| 158 | 13.58758 | 15.38697 | 15.92245 | 14.96567 | 0.789 | 0.91924 | 0.97397 | 0.89407 |
| 162 | 14.01568 | 15.98518 | 16.60225 | 15.53437 | 1.05482 | 1.11221 | 1.22789 | 1.13164 |
| 170 | 13.98842 | 15.59184 | 16.38943 | 15.32323 | 0.80166 | 0.89468 | 1.02614 | 0.90749 |
| 182 | 14.24746 | 16.06713 | 16.92458 | 15.74639 | 0.86495 | 0.98239 | 1.09571 | 0.98102 |
| 194 | 14.01568 | 15.93601 | 16.87912 | 15.61027 | 0.77213 | 0.81398 | 0.88005 | 0.82205 |
| 205 | 14.35653 | 16.48506 | 17.42048 | 16.08736 | 0.95355 | 1.0245 | 1.06441 | 1.01415 |
| 215 | 14.28384 | 16.23185 | 17.21065 | 15.90878 | 0.83212 | 0.91345 | 1.03521 | 0.92693 |
| 230 | 14.13246 | 15.79633 | 17.08623 | 15.67167 | 0.80787 | 0.90085 | 1.00182 | 0.90351 |
| 255 | 13.96115 | 15.21488 | 16.95558 | 15.3772 | 0.77634 | 0.87714 | 0.96353 | 0.87234 |
| 284 | 13.88207 | 15.92236 | 17.19319 | 15.66587 | 0.96621 | 0.96134 | 0.99484 | 0.97413 |
| 301 | 14.53105 | 16.791 | 17.82753 | 16.38319 | 1.03794 | 1.0666 | 1.07484 | 1.05979 |
| 313 | 14.771 | 17.4657 | 18.6003 | 16.94567 | 1.45565 | 1.42447 | 1.38442 | 1.42151 |
| 331 | 14.83645 | 17.16249 | 18.51145 | 16.8368 | 0.94933 | 0.98239 | 1.00179 | 0.97784 |
| 349 | 14.65921 | 16.76368 | 18.36062 | 16.5945 | 0.7004 | 0.81749 | 0.83831 | 0.7854 |
| 362 | 14.41651 | 16.40132 | 18.11346 | 16.31043 | 0.78132 | 0.83659 | 0.85169 | 0.8232 |
| 375 | 14.20864 | 16.15823 | 17.87259 | 16.07982 | 0.82158 | 0.86237 | 0.87238 | 0.85211 |
| 388 | 14.13285 | 15.78132 | 17.58231 | 15.83216 | 0.76938 | 0.82917 | 0.84128 | 0.81328 |
| 397 | 13.85753 | 15.46618 | 17.17253 | 15.49875 | 0.71728 | 0.76486 | 0.76874 | 0.75029 |
|  |  |  |  |  |  |  |  |  |
|  |  |  |  |  |  |  |  |  |
|  |  |  |  |  |  |  |  |  |
|  |  |  |  |  |  |  |  |  |
|  |  |  |  |  |  |  |  |  |
|  |  |  |  |  |  |  |  |  |
|  |  |  |  |  |  |  |  |  |
|  |  |  |  |  |  |  |  |  |

**Effect of temperature on the water content of high clay-content shale**

**5℃-100%RH**

| **Absorption time (hours)** | **Water content of the experiment group (Shale) (%)** | | | | **Water content of the control group (quartz) (%)** | | | |
| --- | --- | --- | --- | --- | --- | --- | --- | --- |
|  | **Sample 1** | **Sample 2** | **Sample 3** | **Average** | **Sample 1** | **Sample 2** | **Sample 3** | **Average** |
| 0 | 0.09677 | 0.13801 | 0.12048 | 0.11842 | 0.08337 | 0.09119 | 0.2506 | 0.14172 |
| 17 | 2.63998 | 3.68185 | 3.6361 | 3.31931 | 0.71201 | 0.6383 | 0.67186 | 0.67406 |
| 29 | 4.14965 | 5.37244 | 5.88668 | 5.13626 | 0.80439 | 0.92228 | 0.87061 | 0.86576 |
| 47 | 6.70254 | 7.51402 | 7.46738 | 7.22798 | 0.79989 | 0.9327 | 1.02832 | 0.9203 |
| 52 | 7.48253 | 8.06359 | 7.84328 | 7.79647 | 0.93057 | 1.01086 | 1.03264 | 0.99136 |
| 65 | 8.74833 | 9.04443 | 8.59508 | 8.79594 | 1.08154 | 1.15676 | 1.02616 | 1.08815 |
| 76 | 9.77607 | 9.82811 | 9.13965 | 9.58128 | 1.0545 | 1.15676 | 1.10825 | 1.1065 |
| 89 | 10.93348 | 10.64137 | 9.87699 | 10.48395 | 1.26855 | 1.24013 | 1.04992 | 1.1862 |
| 100 | 11.58767 | 11.08004 | 10.40469 | 11.02413 | 1.1266 | 1.04994 | 0.95055 | 1.04236 |
| 113 | 12.34637 | 11.637 | 10.75168 | 11.57835 | 1.29334 | 1.13852 | 1.00888 | 1.14691 |
| 122 | 12.65411 | 11.85633 | 10.90107 | 11.80384 | 1.27306 | 1.09684 | 0.98295 | 1.11762 |
| 136 | 13.54056 | 12.37879 | 11.21914 | 12.3795 | 1.29108 | 1.14894 | 0.98079 | 1.14027 |
| 148 | 13.93733 | 12.78542 | 11.37818 | 12.70031 | 1.37445 | 1.16979 | 1.02184 | 1.18869 |
| 161 | 14.33991 | 13.15015 | 11.57817 | 13.02274 | 1.23926 | 1.26618 | 1.05856 | 1.188 |
| 172 | 14.65926 | 13.29309 | 11.68902 | 13.21379 | 1.32263 | 1.16457 | 1.02184 | 1.16968 |
| 191 | 15.27861 | 13.49271 | 11.8095 | 13.52694 | 1.33389 | 1.07078 | 1.01968 | 1.14145 |
| 213 | 16.11086 | 13.85251 | 12.12034 | 14.0279 | 1.38346 | 1.20887 | 1.14714 | 1.24649 |
| 221 | 16.3857 | 13.90427 | 12.30829 | 14.19942 | 1.35868 | 1.18021 | 1.09961 | 1.21283 |
| 243 | 16.95473 | 13.94123 | 12.43118 | 14.44238 | 1.01169 | 0.98481 | 0.89654 | 0.96434 |
| 261 | 17.35344 | 14.03981 | 12.31792 | 14.57039 | 1.04774 | 1.18281 | 0.97647 | 1.06901 |
| 269 | 17.37086 | 13.89934 | 12.2866 | 14.51893 | 1.00718 | 1.07078 | 0.98079 | 1.01959 |
| 293 | 17.69021 | 13.85991 | 12.36612 | 14.63874 | 1.06126 | 1.09423 | 0.96783 | 1.04111 |
| 305 | 17.9573 | 13.99052 | 12.37816 | 14.77533 | 1.15138 | 1.19063 | 1.04776 | 1.12992 |
| 317 | 18.06763 | 13.98806 | 12.35166 | 14.80245 | 1.08379 | 1.12289 | 0.98295 | 1.06321 |
| 341 | 18.7102 | 14.25668 | 12.33961 | 15.10216 | 1.13336 | 1.10986 | 1.05424 | 1.09916 |
| 364 | 19.20568 | 14.21478 | 12.39021 | 15.27023 | 1.11759 | 1.10205 | 1.01104 | 1.07689 |
| 387 | 19.45729 | 14.18521 | 12.42636 | 15.35629 | 1.06802 | 1.00565 | 0.95919 | 1.01095 |
| 406 | 19.74955 | 14.33061 | 12.3878 | 15.48932 | 1.07478 | 1.08642 | 1.01536 | 1.05885 |
| 411 | 20.13471 | 14.31336 | 12.47937 | 15.64248 | 0.96437 | 0.94312 | 0.89438 | 0.93396 |
|  |  |  |  |  |  |  |  |  |

**10℃-100%RH**

| **Absorption time (hours)** | **Water content of the experiment group (Shale) (%)** | | | | **Water content of the control group (quartz) (%)** | | | |
| --- | --- | --- | --- | --- | --- | --- | --- | --- |
|  | **Sample 1** | **Sample 2** | **Sample 3** | **Average** | **Sample 1** | **Sample 2** | **Sample 3** | **Average** |
| 0 | 0.10263 | 0.09982 | 0.12521 | 0.10285 | 0.10285 | 0.13127 | 0.11223 | 0.11545 |
| 5 | 2.96514 | 3.87587 | 2.7715 | 3.20417 | 1.08565 | 1.45205 | 1.07748 | 1.20506 |
| 18 | 6.18538 | 7.19803 | 6.5997 | 6.66104 | 1.8915 | 3.03116 | 2.17492 | 2.36586 |
| 29 | 6.94383 | 8.92702 | 7.16707 | 7.67931 | 1.41966 | 2.26363 | 1.76603 | 1.81644 |
| 42 | 8.14277 | 9.8797 | 9.0188 | 9.01376 | 1.22409 | 1.75473 | 1.30214 | 1.42699 |
| 53 | 9.63112 | 10.41979 | 9.71574 | 9.92222 | 1.14914 | 1.54076 | 1.26741 | 1.3191 |
| 66 | 10.62899 | 12.39834 | 10.45825 | 11.16186 | 1.11596 | 1.17673 | 1.25438 | 1.18236 |
| 77 | 11.47051 | 12.06666 | 10.54409 | 11.36042 | 0.97581 | 1.0604 | 1.12393 | 1.05338 |
| 90 | 11.53771 | 12.4045 | 12.0029 | 11.9817 | 0.98304 | 1.07287 | 1.15004 | 1.06865 |
| 101 | 12.48322 | 12.50702 | 11.89951 | 12.29658 | 0.9599 | 1.18088 | 1.08038 | 1.07372 |
| 114 | 14.46624 | 13.09527 | 12.98433 | 13.51528 | 1.21257 | 1.44998 | 1.39031 | 1.35095 |
| 125 | 15.70299 | 13.90145 | 14.35954 | 14.65466 | 1.53726 | 1.73625 | 1.74451 | 1.67267 |
| 138 | 17.15597 | 14.4509 | 15.50648 | 15.70445 | 1.71751 | 1.66431 | 1.9122 | 1.76467 |
| 162 | 16.27793 | 12.65631 | 14.03454 | 14.32293 | 0.72502 | 0.78519 | 0.72056 | 0.74359 |
| 173 | 16.65414 | 13.40776 | 14.00256 | 14.68815 | 0.88171 | 1.09574 | 0.88691 | 0.95479 |
| 186 | 17.23226 | 14.75053 | 14.43252 | 15.47177 | 1.18374 | 1.38387 | 1.15875 | 1.24212 |
| 197 | 17.1243 | 13.8099 | 14.2228 | 15.05233 | 0.90778 | 0.92297 | 0.87234 | 0.90103 |
| 210 | 17.53614 | 15.08506 | 14.38018 | 15.66713 | 1.20104 | 1.22859 | 1.08619 | 1.17194 |
| 234 | 19.08817 | 14.31316 | 14.97328 | 16.12487 | 1.00763 | 1.08743 | 0.90148 | 0.99884 |
| 269 | 19.77162 | 13.48004 | 14.84927 | 16.03364 | 0.91213 | 1.02711 | 0.92915 | 0.95613 |
| 282 | 19.54438 | 12.97983 | 14.57658 | 15.70026 | 0.73083 | 0.78728 | 0.75416 | 0.75742 |
| 306 | 19.72064 | 12.5991 | 14.58448 | 15.63474 | 0.81647 | 0.74756 | 0.73517 | 0.7664 |
| 367 | 19.94698 | 12.31409 | 14.64766 | 15.63624 | 0.75988 | 0.81027 | 0.75854 | 0.77623 |
| 391 | 20.17217 | 12.43938 | 14.69183 | 15.7678 | 0.78746 | 0.84995 | 0.78774 | 0.80838 |
| 415 | 18.6885 | 13.10741 | 15.19227 | 15.66273 | 0.786 | 0.8583 | 0.78336 | 0.80922 |
| 428 | 18.31248 | 12.7868 | 15.45529 | 15.51819 | 0.76278 | 0.8583 | 0.79066 | 0.80391 |
|  |  |  |  |  |  |  |  |  |
|  |  |  |  |  |  |  |  |  |

**20℃-100%RH**

| **Absorption time (hours)** | **Water content of the experiment group (Shale) (%)** | | | | **Water content of the control group (quartz) (%)** | | | |
| --- | --- | --- | --- | --- | --- | --- | --- | --- |
|  | **Sample 1** | **Sample 2** | **Sample 3** | **Average** | **Sample 1** | **Sample 2** | **Sample 3** | **Average** |
| 0 | 0.18885 | 0.18716 | 0.26948 | 0.21517 | 0.14492 | 0.10183 | 0.1517 | 0.13282 |
| 13 | 5.32814 | 6.24165 | 5.78754 | 5.78578 | 0.85651 | 0.89117 | 0.94614 | 0.89794 |
| 25 | 7.82861 | 9.49769 | 8.82285 | 8.71638 | 0.75947 | 0.82451 | 0.94266 | 0.84221 |
| 37 | 9.22472 | 10.90172 | 10.46758 | 10.19801 | 0.88605 | 0.75785 | 0.83135 | 0.82508 |
| 49 | 10.22818 | 11.78948 | 11.38706 | 11.13491 | 0.68352 | 0.76135 | 0.86614 | 0.77034 |
| 64 | 11.23709 | 13.07332 | 12.78383 | 12.36475 | 0.73837 | 0.83854 | 0.91831 | 0.83174 |
| 74 | 11.59157 | 13.13069 | 13.09584 | 12.60603 | 0.71728 | 0.7789 | 0.9044 | 0.80019 |
| 85 | 12.04967 | 13.59779 | 13.68058 | 13.10935 | 0.66664 | 0.75083 | 0.91136 | 0.77628 |
| 98 | 12.20237 | 13.66334 | 13.97399 | 13.2799 | 0.64977 | 0.71574 | 0.81744 | 0.72765 |
| 108 | 12.43142 | 13.8327 | 14.27773 | 13.51395 | 0.67086 | 0.75083 | 0.86614 | 0.76261 |
| 122 | 12.6223 | 13.8846 | 14.54221 | 13.68303 | 0.64555 | 0.73679 | 0.83831 | 0.74022 |
| 134 | 12.77227 | 14.29434 | 14.79842 | 13.95501 | 0.69196 | 0.78241 | 0.83135 | 0.76857 |
| 146 | 13.18674 | 14.47189 | 15.06083 | 14.23982 | 0.68774 | 0.821 | 0.89744 | 0.80206 |
| 158 | 13.58758 | 15.38697 | 15.92245 | 14.96567 | 0.789 | 0.91924 | 0.97397 | 0.89407 |
| 162 | 14.01568 | 15.98518 | 16.60225 | 15.53437 | 1.05482 | 1.11221 | 1.22789 | 1.13164 |
| 170 | 13.98842 | 15.59184 | 16.38943 | 15.32323 | 0.80166 | 0.89468 | 1.02614 | 0.90749 |
| 182 | 14.24746 | 16.06713 | 16.92458 | 15.74639 | 0.86495 | 0.98239 | 1.09571 | 0.98102 |
| 194 | 14.01568 | 15.93601 | 16.87912 | 15.61027 | 0.77213 | 0.81398 | 0.88005 | 0.82205 |
| 205 | 14.35653 | 16.48506 | 17.42048 | 16.08736 | 0.95355 | 1.0245 | 1.06441 | 1.01415 |
| 215 | 14.28384 | 16.23185 | 17.21065 | 15.90878 | 0.83212 | 0.91345 | 1.03521 | 0.92693 |
| 230 | 14.13246 | 15.79633 | 17.08623 | 15.67167 | 0.80787 | 0.90085 | 1.00182 | 0.90351 |
| 255 | 13.96115 | 15.21488 | 16.95558 | 15.3772 | 0.77634 | 0.87714 | 0.96353 | 0.87234 |
| 284 | 13.88207 | 15.92236 | 17.19319 | 15.66587 | 0.96621 | 0.96134 | 0.99484 | 0.97413 |
| 301 | 14.53105 | 16.791 | 17.82753 | 16.38319 | 1.03794 | 1.0666 | 1.07484 | 1.05979 |
| 313 | 14.771 | 17.4657 | 18.6003 | 16.94567 | 1.45565 | 1.42447 | 1.38442 | 1.42151 |
| 331 | 14.83645 | 17.16249 | 18.51145 | 16.8368 | 0.94933 | 0.98239 | 1.00179 | 0.97784 |
| 349 | 14.65921 | 16.76368 | 18.36062 | 16.5945 | 0.7004 | 0.81749 | 0.83831 | 0.7854 |
| 362 | 14.41651 | 16.40132 | 18.11346 | 16.31043 | 0.78132 | 0.83659 | 0.85169 | 0.8232 |
| 375 | 14.20864 | 16.15823 | 17.87259 | 16.07982 | 0.82158 | 0.86237 | 0.87238 | 0.85211 |
| 388 | 14.13285 | 15.78132 | 17.58231 | 15.83216 | 0.76938 | 0.82917 | 0.84128 | 0.81328 |
| 397 | 13.85753 | 15.46618 | 17.17253 | 15.49875 | 0.71728 | 0.76486 | 0.76874 | 0.75029 |
|  |  |  |  |  |  |  |  |  |

**30℃-100%RH**

| **Absorption time (hours)** | **Water content of the experiment group (Shale) (%)** | | | | **Water content of the control group (quartz) (%)** | | | |
| --- | --- | --- | --- | --- | --- | --- | --- | --- |
|  | **Sample 1** | **Sample 2** | **Sample 3** | **Average** | **Sample 1** | **Sample 2** | **Sample 3** | **Average** |
| 0 | 0.18885 | 0.18716 | 0.26948 | 0.21517 | 0.14492 | 0.10183 | 0.1517 | 0.13282 |
| 10 | 8.07771 | 7.59557 | 7.86854 | 7.84727 | 1.23421 | 1.6564 | 1.18843 | 1.35968 |
| 19 | 10.10589 | 9.70089 | 10.02092 | 9.94257 | 1.12688 | 1.64523 | 1.20152 | 1.32455 |
| 26 | 10.94845 | 10.4917 | 10.7081 | 10.71608 | 1.03052 | 1.38433 | 0.97205 | 1.12897 |
| 35 | 11.17892 | 11.19601 | 11.59308 | 11.32267 | 0.7556 | 1.16383 | 0.99503 | 0.97149 |
| 47.5 | 11.32975 | 11.67458 | 11.4186 | 11.47431 | 0.77049 | 1.09645 | 0.90306 | 0.92333 |
| 59.5 | 11.71715 | 11.66082 | 11.68547 | 11.68781 | 1.23051 | 1.20124 | 1.02785 | 1.1532 |
| 74 | 12.07504 | 11.97804 | 12.03203 | 12.02837 | 0.93033 | 0.96904 | 0.70888 | 0.86941 |
| 84 | 11.70153 | 12.08733 | 11.76269 | 11.85052 | 0.72582 | 1.00278 | 0.71547 | 0.81469 |
| 98 | 11.85972 | 11.94384 | 12.04695 | 11.95017 | 0.6774 | 0.84144 | 0.8373 | 0.78538 |
| 109.5 | 11.67252 | 12.14188 | 11.79483 | 11.86974 | 0.59166 | 0.52918 | 0.61653 | 0.57912 |
| 122.5 | 11.50702 | 11.69005 | 11.64467 | 11.61391 | 0.77421 | 0.77381 | 0.92607 | 0.8247 |
| 145.5 | 11.87752 | 12.39853 | 12.07465 | 12.1169 | 0.81513 | 0.65346 | 1.04097 | 0.83652 |
| 158.5 | 12.19443 | 12.42299 | 12.22108 | 12.2795 | 1.06389 | 0.78885 | 1.06065 | 0.97113 |
| 187.5 | 12.42569 | 12.59116 | 12.48321 | 12.50002 | 0.85231 | 0.65723 | 0.99503 | 0.83486 |
