# Supplementary material for: Hygroscopic effect of high clay-content shale under temperature and humidity conditions and its impact on mechanical properties
Source: PLoS One. 2025 Mar 7;20(3):e0319672. doi: 10.1371/journal.pone.0319672 (PMC11888144; doi:10.1371/journal.pone.0319672)
Supplement: S7 Table — (DOCX) [file pone.0319672.s007.docx]

**S7 Tab.** **Water content, uniaxial compressive strength, and elastic modulus of reconstructed shale**

| **NO.** | **Water content (%)** | **Uniaxial compressive strength (MPa)** | **Elastic modulus (MPa)** |
| --- | --- | --- | --- |
| 1 | 1.20 | 2.35 | 74 |
| 2 | 3.11 | 2.26 | 70 |
| 3 | 3.28 | 2.16 | 72 |
| 4 | 4.98 | 2.09 | 68 |
| 5 | 5.18 | 1.96 | 65 |
| 6 | 7.95 | 1.82 | 62 |
| 7 | 9.53 | 1.72 | 58 |
| 8 | 9.85 | 1.62 | 55 |
| 9 | 11.23 | 1.51 | 50 |
| 10 | 11.58 | 1.33 | 53 |
| 11 | 13.25 | 1.18 | 48 |
| 12 | 14.41 | 1.2 | 45 |
| 13 | 17.01 | 1.06 | 38 |
| 14 | 18.64 | 0.87 | 33 |
| 15 | 20.64 | 0.56 | 21.2 |
| 16 | 22.05 | 0.25 | 20.6 |
